# Recent developments in immunotherapy of cancers caused by human papillomaviruses

**DOI:** 10.1111/imm.13285

**Published:** 2020-12-06

**Authors:** Elham Fakhr, Živa Modic, Angel Cid‐Arregui

**Affiliations:** ^1^ Targeted Tumor Vaccines German Cancer Research Center (DKFZ) Heidelberg Germany; ^2^ Registered at Faculty of Biosciences Heidelberg University Heidelberg Germany; ^3^ Present address: Department of Experimental Oncology Institute of Oncology Ljubljana Ljubljana Slovenia

**Keywords:** cervical cancer, Engineered TCR T cells, human papillomavirus, immunotherapy

## Abstract

A subset of oncogenic human papillomaviruses (HPVs) is the main cause of genital cancers, most importantly cervical cancer and an increasing number of head and neck cancers. Despite the availability of prophylactic vaccines against the most prevalent oncogenic HPV types, HPV‐induced malignancies are still a major health and economic burden. Besides conventional treatment with surgery, chemotherapy and radiation, immunotherapy is emerging as an efficient adjuvant option. Here, we review relevant studies and ongoing clinical trials using immune checkpoint inhibitors, therapeutic vaccines, gene editing approaches and adoptive T cell therapies, with special focus on engineered TCR T cells, which are showing encouraging results and could lead to significant improvement in the treatment of HPV+‐infected cancer patients.

## Graphical abstract

Most therapeutic vaccines targeting oncogenic HPVs are still in early clinical studies. Antibodies against immune checkpoint inhibitors have sown some success. A combination of different immunotherapeutic approaches will be needed to overcome current limitations in the immunotherapy of HPV‐associated cancers.

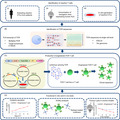

**Abbreviations**
AP‐1: Activator protein 1
ATC: Adoptive T cell therapy
CAR: Chimeric antigen receptor
CFU: Colony‐forming unit
CIN: Cervical intraepithelial neoplasia
CRISPR/Cas9: Clustered regularly interspaced short palindromic repeats/Cas9 protein
CTLA‐4: Cytotoxic T‐lymphocyte‐associated protein 4
CTLs: Cytotoxic T cells
DCs: Dendritic cells
Flt3L: Fms‐like tyrosine kinase‐3 ligand
HLA: Human leucocyte antigen
HPV: Human papillomavirus
HPVST: HPV‐specific T cells
HR: High risk
HSIL: High‐grade squamous intraepithelial neoplasia
IFN‐α: Interferon‐α
IL‐7: Interleukin‐7
LAG‐3: Lymphocyte activation gene‐3
LCR: Long control region
LR: Low risk
MHC: Major histocompatibility complex
MSGV1: murine stem cell virus‐based splice‐gag vector
MVA: Modified vaccinia virus Ankara
NFAT: Nuclear factor of activated T cells
NF‐κB: Nuclear factor ‘kappa‐light‐chain‐enhancer’ of activated B cells
PBMC: Peripheral blood mononuclear cell
PD‐1: Programmed death receptor 1
PD‐L1: PD ligand 1
pMHC: Peptide epitopes loaded on MHC molecules
PSMA: Prostate‐specific membrane antigen
rhGM‐CSF: Recombinant human granulocyte–macrophage colony‐stimulating factor
RTCA: Real‐time cell assays
Scfv: Single‐chain fragment variable
SPR: Surface plasmon resonance
TAA: Tumour‐associated antigen
TALENs: Transcription activator‐like effector nuclease
TCR: T cell receptor
TGF‐β: Transforming growth factor‐β
TILs: Tumour‐infiltrating lymphocytes
TLR: Toll‐like receptor
ZFN: Zinc finger nuclease

## INTRODUCTION

Human papillomaviruses (HPV) are small, non‐enveloped DNA viruses that belong to the family of *Papillomaviridae*, which consists of 52 genera and nearly 200 virus types. The most extensively studied are those of the genus Alphapapillomavirus, which is divided into high‐risk types (HR‐HPVs), most importantly HPV16 and HPV18, classified as carcinogenic (IARC Group 1), and low‐risk HPV types that cause mild dysplasia and benign tumours.^1^, ^2^ The genome of the HPVs is circular, double‐stranded molecule of approximately 8 kb, divided into three regions: long control region (LCR) involved in the regulation of viral gene expression; early (E) region encoding non‐structural proteins needed for viral replication and survival; and finally, late (L) region encoding two structural capsid proteins. Most HR‐HPV infections regress spontaneously; however, in about 10% of cases the immune system fails to clear the infection, which persists and may cause malignant transformation. In such cases, the viral DNA can get randomly integrated into the host genome,^3^, ^4^ usually causing disruption of the E2 gene, which otherwise encodes as a transcriptional repressor of the early gene promoter in the LCR, leading to deregulated expression of the main HR‐HPV oncogenes – E6 and E7.^5^, ^6^, ^7^ These two proteins target several intracellular signalling pathways involved in cell cycle regulation, apoptosis, maintenance of genomic integrity and DNA repair.^8^, ^9^, ^10^, ^11^, ^12^

**Figure 1:**
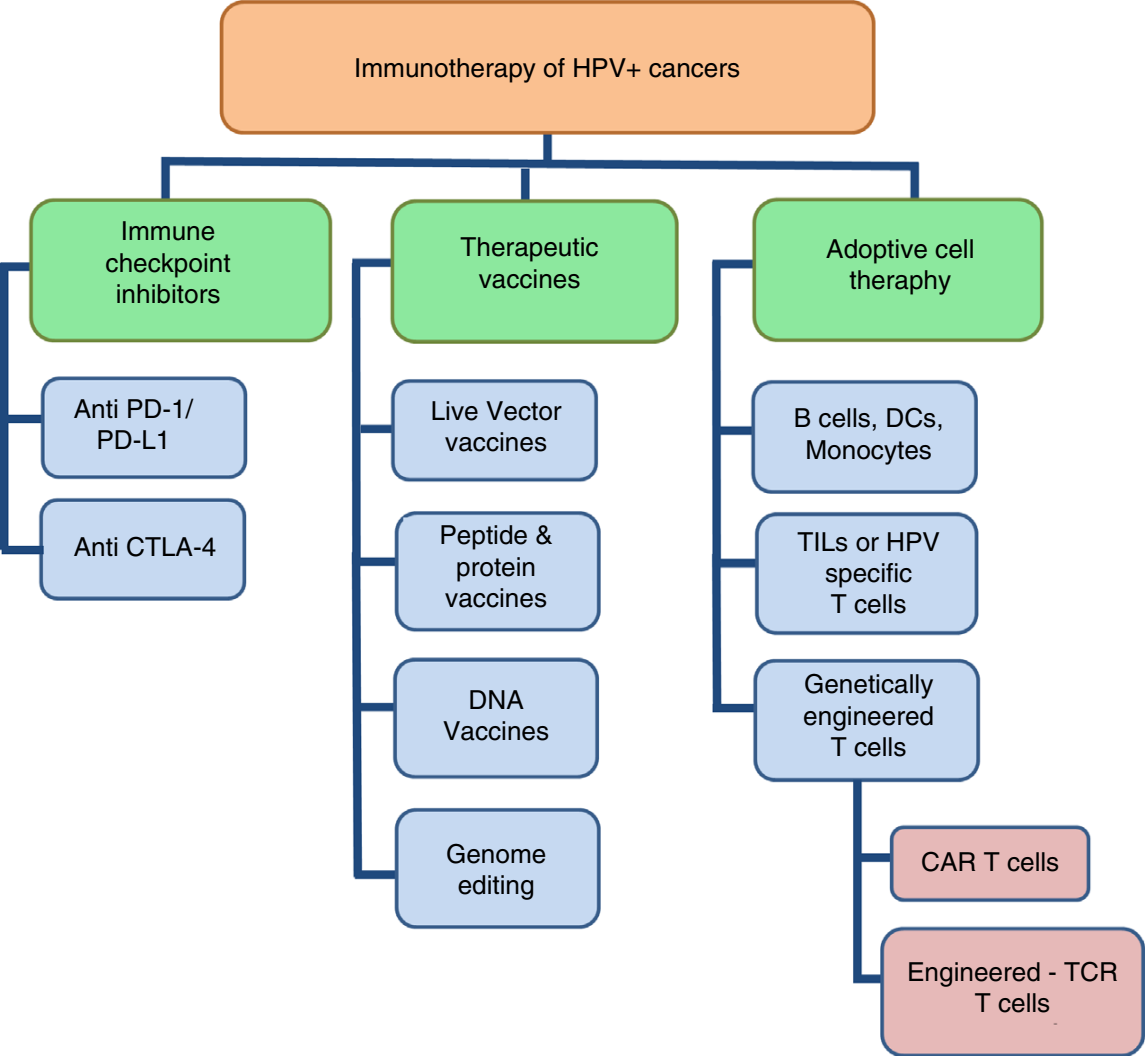
Immunotherapeutic approaches for the treatment of HPV‐associated cancers

According to data from GLOBOCAN 2018,^13^ persistent infection with HR‐HPV types is linked to approximately 4.5% of all cancer cases globally. HR‐HPVs are recognized as the main causative agent of cervical, anal, vulvar, vaginal and penile cancers. Moreover, they are linked to an increasing number of head and neck cancers as well.^14^ Three prophylactic vaccines have been approved to date (quadrivalent Gardasil^®^, nonavalent Gardasil‐9^®^ and bivalent Cervarix^®^) made of virus‐like particles of the most frequent HR‐HPV types, the latter also including two LR‐HPV types that cause genital warts. It has been estimated that the non‐valent vaccine could potentially prevent up to 90% of HPV‐related cancers.^15^ However, there is still a need for effective therapies to treat advanced cases. Cervical cancer is the fourth most common type of cancer and the fourth leading cause of cancer mortality among women worldwide.^13^ Recently, immune‐directed therapies applied to advanced HPV‐positive cancers have shown significant success. Here, we review various immunotherapeutic approaches applied to HPV‐associated cancer patients in clinical trials, with special focus on engineered TCR T cells. Our review follows the flow chart shown in Figure 1.

## IMMUNE CHECKPOINT INHIBITORS

Inhibitors targeting two negative immune regulatory pathways of T cells, the programmed death receptor 1 (PD‐1) and its ligand (PD‐L1) and the cytotoxic T‐lymphocyte‐associated protein 4 (CTLA‐4), have been assessed in clinical trials as having high therapeutic potential.^16^ There are several ongoing clinical trials with anti‐PD‐1/ PD‐L1 and anti‐CTLA‐4. Immune checkpoint inhibitors can be used solely or in combination with chemotherapy, chemoradiation and antiangiogenic agents. Ongoing clinical trials related to anti‐PD‐1/ PD‐L1 and anti‐CTLA‐4 for HPV (+) cancers are summarized in Tables 1 and 2, respectively.

**Table 1:** Ongoing clinical trials of anti‐PD‐1/PD‐L1 for the treatment of HPV‐associated cancers

Agent(s)	Type	Clinical phase	Start date	Completion date	Clinical trial reference
Nivolumab	Anti‐PD‐1	II	May 2015	Mar. 2019^a^	NCT02257528
Nivolumab /ipilimumab /daratumumab /relatlimab	Anti‐PD‐1 /Anti‐CTLA‐4 /Anti‐CD 38 /Anti‐LAG‐3	I/II	Oct. 2015	May 2022	NCT02488759
Nivolumab /cisplatin /radiotherapy	Anti‐PD‐1 /chemotherapy	I/II	Nov. 2017	April 2020	NCT03298893
Nivolumab /cisplatin /radiotherapy	Anti‐PD‐1 /chemotherapy	II	June 2020	Aug. 2023	NCT04282109
Pembrolizumab	Anti‐PD‐1	II	Dec. 2015	Aug. 2023	NCT02628067
Pembrolizumab /paclitaxel/cisplatin/carboplatin/bevacizumab/Placebo	Anti‐PD‐1 /platinum‐based chemotherapy /antiangiogenic agents	III	Oct. 2018	Nov. 2022	NCT03635567
Pembrolizumab /platinum /brachytherapy	Anti‐PD‐1 /chemotherapy /radiotherapy	I	Dec. 2017	Jan. 2019	NCT03144466
Pembrolizumab /cisplatin /brachytherapy	Anti‐PD‐1 /chemotherapy /chemoradiation	II	Jan 2016	May 2021	NCT02635360
Pembrolizumab / paclitaxel/cisplatin/carboplatin /bevacizumab	Anti‐PD‐1/platinum‐based chemotherapy /antiangiogenic agents	II	Sept. 2018	Oct. 2025	NCT03367871
Pembrolizumab /immunomodulatory / cocktail /radiotherapy	Anti‐PD‐1 /vitamin D, aspirin, cyclophosphamide and lansoprazole	II	July 2017	June 2022	NCT03192059
AGEN2034 (RebmAb‐700)	Anti‐PD‐1	I/II	April 2017	Sept. 2019	NCT03104699
REGN2810 (cemiplimab)	Anti‐PD‐1	I/II	April 2017	Sept. 2019	NCT03257267
Durvalumab /vigil	Anti‐PD‐L1 /cellular immunotherapy	II	June 2016	Jan. 2021^a^	NCT02725489
M7824	Bifunctional antibody targeting TGF‐β and PD‐L1	II	Feb. 2018	Dec. 2023	NCT03427411
Atezolizumab /carboplatin /cyclophosphamide	Anti‐PD‐L1 /chemotherapy	I	Jan. 2017	June 2019^a^	NCT02914470
Atezolizumab /cisplatin/paclitaxel /bevacizumab	Anti‐PD‐L1 /chemotherapy /antiangiogenic agents	III	Sept. 2018	Dec. 2023	NCT03556839
Atezolizumab /bevacizumab	Anti‐PD‐L1 /antiangiogenic agents	II	Mar. 2017	Aug. 2020^a^	NCT02921269

^a^ Active, not recruiting.

**Table 2:** Ongoing clinical trials of CTLA‐4 inhibitors for the treatment of HPV‐associated cancers

Agent(s)	Type	Clinical phase	Start Date	Completion Date	Clinical trial reference
Ipilimumab	Anti‐CTLA‐4	II	Dec. 2012	Dec. 2020^a^	NCT01693783
Ipilimumab /cisplatin /radiation	Anti‐CTLA‐4/chemotherapy	I	Oct. 2012	Mar. 2017^a^	NCT01711515
Ipilimumab /cetuximab /radiotherapy	Anti‐CTLA‐4 /antiangiogenic /radiotherapy	I	Apr. 2013	Oct. 2016^a^	NCT01935921
Tremelimumab /durvalumab	Anti‐CTLA‐4 /anti‐PD‐L1	I	Dec. 2013	Dec. 2020^a^	NCT01975831
Nivolumab /ipilimumab /radiotherapy	Anti‐PD‐1 /Anti‐CTLA‐4 /radiotherapy	II	July 2019	Aug. 2022	NCT03799445
Tremelimumab /durvalumab /vinorelbine	Anti‐CTLA‐4 /Anti‐PD‐L1 /chemotherapy	I/II	Jun 2018	Aug. 2023	NCT03518606
Tremelimumab /durvalumab /radiotherapy	Anti‐CTLA‐4/anti‐PD‐L1 /radiotherapy	I	July 2018	Oct. 2020	NCT03452332

^a^ Active, not recruiting.

### Anti‐PD‐1/PD‐L1 monoclonal antibodies

Nivolumab, a fully human antibody against PD‐1, has been tested as a monotherapy in patients suffering from recurrent or metastatic cervical cancer (NCT02257528). The results of this study showed that solely administration of nivolumab was safe but had low antitumour effect.^17^ However, the initial results of another phase I/ II clinical study (NCT02488759) have shown that the efficacy of nivolumab monotherapy is promising in metastatic vaginal, vulvar and cervical cancers.^18^ The efficiency and safety of nivolumab plus chemoradiation (NCT03298893) and in combination with paclitaxel (NCT04282109) are being studied in patients suffering from locally advanced cervical cancer or recurrent head and neck squamous cell carcinoma (HNSCC), respectively. The results of these studies have not been reported yet, but the combo therapies are expected to be more effective than nivolumab alone.

Pembrolizumab is a humanized antibody targeting PD‐1 that has shown promising outcomes in a clinical phase II study as monotherapy of metastatic solid tumours including cervical and vulvar cancers. The antitumour activity of pembrolizumab in patients with PD‐L1‐positive advanced cervical cancer was stable, and the toxicity was manageable (NCT02628067).^19^ Based on these findings, the US Food and Drug Administration has accelerated the approval of pembrolizumab for PD‐L1‐positive advanced cervical cancer suffering from progression in the course of chemotherapy or afterwards. Furthermore, in a phase III clinical trial, pembrolizumab monotherapy also showed encouraging results in PD‐L1‐positive recurrent or metastatic HNSCC. The published results also support that pembrolizumab in combination with 5‐fluorouracil and platinum chemotherapy can be considered as a first‐line treatment for HNSCC.^20^

In yet another phase III clinical trial, the safety and antitumour activity of pembrolizumab is being studied in combination with platinum‐based chemotherapy in patients with recurrent, persistent or metastatic cervical cancer (NCT03635567). The study is planned to be completed on November 2022, and it is expected to provide valuable information on the suitability of this therapeutic regime as the first‐line treatment of recurrent or metastatic cervical cancer. In a phase II study, durvalumab, anti‐PD‐L1, is being combined with a personalized cellular immunotherapy called vigil in advanced cancers including cervical carcinomas (NCT02725489). Vigil consists of irradiated autologous tumour cells genetically engineered to suppress transforming growth factor‐β1 and transforming growth factor‐β2 (TGF‐β1 and TGF‐β2) and express recombinant human granulocyte–macrophage colony‐stimulating factor (rhGM‐CSF). Moreover, in another phase II study, M7824, a bifunctional antibody targeting TGF‐β and PD‐L1, is being studied in HPV‐associated cancers (NCT03427411). A combinational therapy of chemotherapeutic drugs, carboplatin–cyclophosphamide and atezolizumab (anti‐PD‐L1), is under study in a phase I study (NCT02914470). Further, in an active, recruiting phase III clinical trial, atezolizumab is combined with cisplatin/paclitaxel/bevacizumab, a standard treatment of stage IVB cervical carcinoma (NCT03556839).^21^ In addition, in a running phase II clinical study, bevacizumab is combined with atezolizumab in advanced cervical cancers. Interestingly, this study includes intratumoral and peripheral T cell receptor (TCR) clonality assessment by TCR sequencing (NCT02921269).

### Anti‐CTLA‐4 monoclonal antibodies

Upon ligand binding on activated T cells, CTLA‐4 down‐modulates their cytotoxic responses. It has been shown recently that single nucleotide polymorphisms in the promoter region of the CTLA‐4 gene correlate with higher susceptibility to various types of malignancies including cervical cancer.^22^ Ipilimumab, a fully human anti‐CTLA‐4 monoclonal antibody, is being tested in a phase II study to assess its efficacy in patients with recurrent or metastatic cervical cancer (NCT01693783). Initial results on 34 evaluated patients showed three partial responses and eight cases with stable disease, while 23 patients had progression of disease. Moreover, ipilimumab was well tolerated in cervical cancer patients and promoted immune activation.^23^ Another phase I study recruits patients with locally advanced cervical cancer to receive chemotherapy with cisplatin and chemoradiation followed by administration of ipilimumab. This study will measure HPV‐specific T cells and differential activated T cell responses based on the HLA‐subtype A*0201 (NCT01711515). According to an initial report on 2017, ipilimumab was efficient and well tolerated.^24^ Safety and dosage of ipilimumab in combination with cetuximab is also being studied in patients with stage III/IVB head and neck cancer (NCT01935921).

In yet another approach, anti‐PD‐1/PD‐L1 and anti‐CTLA‐4 antibodies are used simultaneously. An active phase I study was designed to evaluate the safety and tolerance of tremelimumab, anti‐CTLA‐4 antibody and durvalumab in various advanced solid tumours including cervical cancer (NCT01975831). Preliminary results showed that this combination is safe and has clinical activity.^25^ Combination of nivolumab, ipilimumab and radiotherapy is also being evaluated in HPV‐positive head and neck cancer (NCT03799445). Combinational therapy with durvalumab, tremelimumab and vinorelbine (chemotherapeutic drug that inhibits mitosis through interaction with tubulin) is being evaluated in a phase I/II study for advanced solid tumours including cervical cancer (NCT03518606). Tremelimumab combined with durvalumab and radiotherapy is being studied for the treatment of cervical, vaginal or vulvar cancers (NCT03452332).

## THERAPEUTIC VACCINES

Most therapeutic HPV vaccines target the two major oncoproteins of HPV16 and HPV18, E6 and E7, which are constitutively expressed by tumour cells.^26^ A number of strategies have been applied to develop therapeutic vaccines using vectors, peptides/proteins, DNA and genome editing tools. Vector, peptide and protein vaccines are mainly HPV16‐specific, while DNA vaccines and genome editing tools are mostly polyvalent vaccines targeting E6 and E7 genes of both HPV16 and HPV18. Table 3 summarizes recent clinical studies using vaccines.

**Table 3:** Ongoing clinical trials with therapeutic vaccines for the treatment of HPV‐associated cancers

Agent	Type	HPV target	Clinical phase	Start Date	Completion Date	Reference
ADXS11‐001	Bacterial vector	HPV16 E7	II	May 2013	Oct 2018^a^	NCT01266460
ADXS11‐001 /placebo	Bacterial vector	HPV16 E7	III	Sept. 2016	Oct. 2024^a^	NCT02853604
ADXS 11‐001	Bacterial vector	HPV16 E7	II	Dec. 2013	Aug. 2023^a^	NCT02002182
ADXS11‐001/5‐fluorouracil/mitomycin	Bacterial vector/ chemotherapy	HPV16 E7	I/II	Feb. 2013	Feb. 2018	NCT01671488
ADXS11‐001	Bacterial vector	HPV16 E7	I/II	Jan. 2015	July 2018	NCT02164461
ADXS11‐001 /durvalumab	Bacterial vector /anti‐PD‐L1	HPV16 E7	I/II	Apr. 2015	Dec. 2019	NCT02291055
TG4001/avelumab	Viral vector/anti‐PD‐L1	HPV‐16 E6/E7	I/II	Sept. 2017	Dec. 2021	NCT03260023
Vvax 001	RNA replicon vaccine	HPV‐16 E6/E7	I	Jan. 2017	Nov. 2017	NCT03141463
TA‐HPV /pNGVL4a‐Sig/E7(detox)/HSP70 /imiquimod	Viral vector /DNA vaccine /immune response modifier	HPV‐16/18 E6/E7	I	Nov. 2008	Dec. 2021	NCT00788164
PRGN‐2009 /M7824	Viral vector /bifunctional antibody targeting TGF‐β and PD‐L1	HPV‐16 E6/E7	I/II	Aug. 2020	Oct. 2023	NCT04432597
DPX‐E7	Peptide vaccine	HPV16‐E7(11‐19)	I/II	Dec. 2016	May 2023	NCT02865135
ISA 101 /Nivolumab	Peptide vaccine/anti‐PD‐1	HPV‐16 E6/E7	II	Dec. 2015	Dec. 2019^a^	NCT02426892
ISA 101 /carboplatin/paclitaxel /bevacizumab	Peptide vaccine /chemotherapy /antiangiogenic	HPV‐16 E6/E7	I/II	Sept. 2013	Aug. 2018	NCT02128126
PepCan	Peptide vaccine	HPV‐16 E6	II	Oct. 2015	Aug. 2020	NCT02481414
Hespecta	Peptide vaccine	HPV16 E6	I	Mar. 2015	Dec. 2017	NCT02821494
HPV16 E7 peptide, synthetic HPV16 E6 peptide	Peptide vaccine	HPV16 E6/E7	I	Jan. 2003	Apr. 2015	NCT00019110
TA‐CIN	Protein vaccine	HPV16 L2/ E6/E7	I	Apr. 2019	Nov. 2022	NCT02405221
TVGV‐1	Protein vaccine	HPV16 E7	II	Nov. 2015	Sept. 2018^a^	NCT02576561
GX‐118E	DNA vaccine	HPV16/18 E6/E7	II	July 2014	Mar. 2016	NCT02139267
GX‐118E/placebo	DNA vaccine	HPV16/18 E6/E7	II	Aug. 2015	Aug. 2018^a^	NCT02596243
GX‐118E/GX‐I7/imiquimod	DNA vaccine /IL‐7 /antigenital warts	HPV16/18 E6/E7	Interventional clinical trial	May 2017	Oct. 2018	NCT03206138
VGX‐3100 /INO‐9012	DNA vaccine /DNA vaccine encoding IL‐12	HPV16/18 E6/E7	I/IIa	May 2014	Sept. 2017	NCT02172911
VGX‐3100 /placebo	DNA vaccine	HPV16/18 E6/E7	III	June 2017	Apr. 2021^a^	NCT03185013
VB10.16	DNA vaccine	HPV16 E6	I/II	Aug. 2015	Jan. 2019	NCT02529930
pnGVL4a‐CRT/E7 (Detox)	DNA vaccine	HPV16 E7	I	Oct. 2009	July 2018	NCT00988559
pnGVL4a‐CRT/E7 (Detox)/ cyclophosphamide	DNA vaccine / chemotherapy	HPV16 E7	I	Dec. 2011	Nov. 2018	NCT01493154
ZFN‐603/ZFN‐758	Zinc finger nuclease	HPV16/18 E7	I	Dec. 2016	July. 2017^a^	NCT02800369
TALEN/CRISPR/Cas9	Genome editing tools	HPV16/18 E6/E7	I	Jan. 2018	Jan. 2019	NCT03057912

^a^ Active, not recruiting

### Vector vaccines

Certain recombinant bacterial and viral vectors can replicate inside the cells and promote antigen presentation.^27^ ADXS11‐001 is an attenuated live *Listeria* *monocytogenes* (LM) encoding a HPV16 E7 oncoprotein linked to LM listeriolysin O, which has been evaluated in several clinical trials for the treatment of HPV16(+) cancers including cervical cancer. ADXS11‐001 monotherapy has shown promising outcomes in terms of safety and efficacy in recurrent cervical cancer patients (NCT01266460). The first stage of these two‐stage phase II clinical study showed 12‐month overall survival for 38.5% of the patients.^28^ In the second stage, 34.9% had 12‐month overall survival rate and these outcomes encourage further clinical studies of ADXS11‐001.^29^ In a phase III clinical trial, this vaccine was also compared with placebo in disease‐free interval of patients with high‐risk locally advanced cervical cancer (NCT02853604). ADXS11‐001 is also being studied in HPV‐positive head and neck cancers to evaluate whether it can simulate the immune system prior to surgery (NCT02002182). A recent completed phase I/II trial with ADXS11‐001 showed that 1 × 10^9^ colony‐forming units (CFU) could be safely administered to patients with advanced anal cancer and that the vaccine, solely or in combination with cisplatin, had antitumour activity (NCT01671488).^30^ Another phase I/II study showed the safety of a higher dose of ADXS11‐001 (1 × 10^10^ CFU) in cervical cancer patients at different stages (NCT02164461).^31^ In a phase I/II clinical trial, the same vaccine was administered alone or in combination with durvalumab in previously treated locally advanced or metastatic cervical or head and neck cancer patients (NCT02291055).^32^ ADXS11‐001, as monotherapy or in combination, has shown encouraging outcomes, and the results of more clinical studies can pave its path to be approved as an alternative therapy for HPV‐positive cancer patients.

Modified vaccinia virus Ankara (MVA) vectors encoding E6 or E7 oncoprotein of HPV16 or HPV18 have been shown to drive HPV‐specific cytotoxic T‐lymphocyte (CTL) responses. TG4001 is a MVA vector expressing HPV16 E6/E7 and IL‐2, which is currently being evaluated in a phase I/II trial (NCT03260023) in HPV16(+) recurrent or metastatic cancers in combination with avelumab, a humanized IgG1 monoclonal antibody targeting human PD‐L1. Interim results suggest that administration of TG4001 plus avelumab in HPV16+ cancer patients is safe and induces antitumour activity.^33^

Vvax001 is an RNA replicon derived from Semliki Forest virus encoding a fusion of the HPV16 E6 and E7 proteins that is being tested for the first time in humans to evaluate its efficiency and safety in advanced cervical cancer in a running clinical study (NCT03141463).

TA‐HPV is a live recombinant vaccina virus expressing modified E6/E7 genes of HPV16/18. Based on the result of a phase I clinical study, TA‐HPV in combination with pNGVL4a‐Sig/E7 (detox)/HSP70 DNA vaccine^34^ and imiquimod is well tolerated, and can induce effector immune response in HPV16+ CIN III patients (NCT00788164).^35^

PRGN‐2009 is a novel gorilla adenovirus including multiple E6/E7 epitopes of HPV16 and HPV18. Due to its promising preclinical results,^36^ it is being evaluated in HPV (+) cancer patients (NCT04432597).

### Peptide and protein vaccines

Peptide vaccines use identified MHC class I‐restricted immunogenic epitopes from HPV antigens. DPX‐E7 is a 9‐amino‐acid‐long synthetic peptide, HPV16 E7_11‐19_, packaged into liposomes, freeze‐dried and resuspended in oil. DPX‐E7 efficacy is being tested in an open‐label phase Ib/II trial in HLA‐A*02‐01 patients with HPV16‐associated head and neck, anal and cervical cancers (NCT02865135).

ISA101 (ISA Pharmaceuticals) is a synthetic long peptide (SLP) vaccine consisting of 12 SLPs (25–35 residues each) from the E6 and E7 oncoproteins of HPV16. ISA101 is being studied combined with nivolumab in patients with HPV16‐positive solid tumours in a phase II trial (NCT02426892). Based on the overall response rate and median overall survival, the results showed promising outcomes compared with solo administration of anti‐PD‐1.^37^ In another phase I/II clinical study, ISA 101 is used in combination with pegylated interferon (IFN‐α IIb). Their results showed that there is a significant correlation between overall survival and T cell responses induced by the vaccine.^38^ Hespecta is another vaccine of the same company consisting of two HPV16 E6‐derived SLPs conjugated to a synthetic Toll‐like receptor 2 (TLR2) ligand (Amplivant). Dose–escalation, toxicity and ability to induce HPV16 E6‐specific T cell responses of Hespecta are being tested in a phase I study (NCT02821494).

PepCan is a vaccine consisting of four HPV16 E6 peptides, which was designed as therapy for high‐grade squamous intraepithelial neoplasia (HSIL). It was safe in a phase I study combined with a *Candida* skin test reagent as adjuvant.^39^ The efficacy and safety of PepCan is currently being evaluated in a phase II study in patients with HSIL (NCT02481414).

In contrast to peptide vaccines, protein vaccines include all antigenic epitopes of E6/E7. Yet, they are less immunogenic and induce mainly MHC class II presentation.^40^ However, vaccines using fusion proteins are more immunogenic and promote presentation through MHC I pathway and subsequent activation of CTLs.^41^ TA‐CIN is a vaccine consisting of an HPV16 L2/E6/E7 fusion protein, which is currently being evaluated in a phase I study in patients with HPV16 (+) cervical cancer (NCT02405221). TVGV‐1 is another fusion protein vaccine consisting of peptide sequence of human HPV16 E7 fused to the *Pseudomonas aeruginosa* exotoxin A and an endoplasmic reticulum retention signal (KDEL), combined with GPI‐0100 as adjuvant. Its efficiency and safety are being studied in a phase II trial enrolling HPV16/18 (+) HSIL patients (NCT02576561).

### DNA vaccines

DNA vaccines have been tested in different clinical studies and demonstrated to be safe, but they generally act as poor immunogens.^41^, ^42^ Therefore, strategies to improve the processing and presentation of antigens encoded by DNA vaccines have been developed to improve their immunogenicity. GX‐188E (Genexine, Inc.) is one such vaccine consisting of a tissue plasminogen activator signal sequence, an FMS‐like tyrosine kinase 3 ligand, and shuffled E6 and E7 genes of HPV16 and HPV18. Safety and efficiency of GX‐118E, applied intramuscularly by electroporation, has been studied in different trials in patients with cervical intraepithelial neoplasia II/III (CIN II/III) (NCT02139267, NCT02596243), and its efficacy in a cohort of 64 CIN III patients has been reported recently.^43^ In another interventional study, the efficiency of GX‐188E in combination with GX‐I7, interleukin‐7 (IL‐7) or imiquimod is being tested in CINIII patients (NCT03206138).

VGX‐3100 (Inovio Pharmaceuticals) is another DNA vaccine consisting of plasmids encoding modified E6 and E7 genes of HPV16/HPV18. The efficiency and safety of VGX‐3100 combined with INO‐9012 (a plasmid encoding IL‐12), labelled INO‐3112, was tested in a phase I/IIa trial in patients with recurrent or persistent cervical cancer (NCT02172911). Another phase III trial is active on patients with CIN II or CIN III associated with HPV16/HPV18 (NCT03185013). VB10.1 (Vaccibody AS6) is another DNA vaccine encoding a chimeric protein composed of an HPV16 E6/E7 fusion, a dimerization domain and an MIP‐1α sequence that binds to antigen‐presenting cells. VB10.1 was tested recently in woman with HPV16(+) CIN II/III in a phase I/IIa (NCT02529930) and reported to be safe and induce E6/E7‐specific CD8+ T cell responses.^44^ PNGVL4a‐CRT/E7 (detox) is another promising DNA vaccine that induced robust immune response in HPV16 CIN II/III patients (NCT00988559).^45^ This vaccine encodes calreticulin, which is linked to a detoxified form of HPV16 E7 and has been also evaluated for treating head and neck cancer patients in a phase I clinical study (NCT01493154).

### Genome editing tools

It has been shown recently that genome editing tools such as clustered regularly interspaced short palindromic repeats/Cas9 protein (CRISPR/Cas9), zinc finger nucleases (ZFNs) and transcription activator‐like effector nuclease (TALENs) can decrease tumorigenicity in HPV16/18 in vitro and in vivo models.^46^, ^47^, ^48^ ZFN‐603 and ZFN‐758 can cleave the HPV16/18 E7 oncogene and reduce the expression of E7 leading to apoptosis of tumour cells. They are being evaluated in a phase I clinical study for the treatment of HPV16‐ or HPV18‐positive CIN (NCT02800369). TALEN and CRISPR/Cas9 targeting HPV16 and HPV18 E6/E7 are also being evaluated in a phase I study for the treatment of HPV (+) CIN patients (NCT03057912).

## CELL‐BASED THERAPIES

In cell‐based therapy, DCs, B cells or T cells are isolated from the patient, transduced ex vivo to express or target a specific antigen and infused back to the same patient. Ongoing clinical trials testing adoptive cell therapies for the treatment of cervical cancer are summarized in Table 4.

**Table 4:** Ongoing clinical trials of adoptive cell therapy for the treatment of HPV‐associated cancers

Agent	Type	Clinical phase	Start date	Completion date	Reference number
BVAC‐C	Cell‐based therapy (B cell/monocyte)	I/II	Oct. 2016	Aug. 2020	NCT02866006
HPVST /Cytoxan/fludarabine /nivolumab	HPV‐specific T cells /chemotherapy /anti‐PD‐1	I	Sept. 2015	Oct. 2033	NCT02379520
Tumour‐infiltrating lymphocytes /fludarabine /cyclophosphamide /aldesleukin	Adoptive cell transfer /chemotherapy /IL‐2	II	Apr. 2012	Aug. 2016	NCT01585428
CAR T cell	Anti‐GD2, PSMA, Muc1, mesothelin or other markers positive for cervical cancer	I/II	Nov. 2015	Dec. 2020	NCT03356795
Engineered TCR T cell /fludarabine/cyclophosphamide /aldesleukin	E6 (29‐38)‐reactive TCR /chemotherapy/IL‐2	I/II	Oct. 2014	June 2016	NCT02280811
Engineered TCR T cell /aldesleukin	E6 (29‐38)‐reactive TCR /IL‐2	I	Jan. 2018	May 2019	NCT03197025
Engineered TCR T cell /anti‐PD‐L1	E6 (29‐38)‐reactive TCR	I	Sept. 2018	Aug. 2021	NCT03578406
Engineered TCR T cell /fludarabine/cyclophosphamide /aldesleukin	E7 (11‐19)‐reactive TCR /chemotherapy /IL‐2	I/II	Jan. 2017	Jan. 2026	NCT02858310
Engineered TCR T cell	E7‐reactive TCR	II	Oct. 2019	June 2020	NCT03937791
KITE‐439 /cyclophosphamide /fludarabine	HPV16 E7 T cell receptor engineered T cells /chemotherapy	I	June 2019	Feb. 2037	NCT03912831

Clinical studies using adoptive T cell therapy (ATC) strategies for the treatment of metastatic or recurrent cervical cancer have shown encouraging results. The first study that explored ATC in cervical cancer was performed by Stevanović et al.^49^ on nine patients with HPV18‐ or HPV16‐associated metastatic cervical cancer, who were treated with tumour‐infiltrating lymphocytes (TILs) expanded ex vivo. Ramos et al.^50^ showed that HPV‐specific T cells (called HPVST) derived from patients with HPV (+) cancers could be useful for immunotherapy of HPV‐associated cancers. HPVST have been engineered to be resistant to TGF‐β. In an ongoing phase I clinical trial (NCT02379520), the safety dose and durability of HPVST is being studied in patients with recurrent HPV‐associated cancers in combination with nivolumab. It has been also shown that HPV‐TILs can cause tumour regression in HPV‐associated cancers (NCT01585428).^51^ Nevertheless, the process of isolating and expanding tumour‐specific T cells is very labour and cost‐intensive and not all patients have HPV‐reactive peripheral or infiltrating T cells. Therefore, an ‘off‐the‐shelf’ approach with genetically engineered T cells would be desirable.^52^

### Chimeric antigen receptor (CAR) T cell

CAR T cells are engineered T cells expressing a single‐chain fragment variable (scfv), which can recognize a tumour‐associated antigen (TAA). They also utilize CD3‐related components and costimulatory domains as intracellular signalling machinery. CAR T cells are MHC‐independent and can activate the T cells upon binding the antigens expressed on the surface of the tumour cells.^53^ A phase I/II study (NCT03356795) evaluates the safety and efficacy of CAR T cells in cervical cancer patients whose tumour cells express TAAs such as GD2, PSMA, Muc1 or mesothelin. In this study, T cells are isolated from peripheral blood mononuclear cells (PBMCs) obtained through apheresis from cervical cancer patients positive for the respective TAAs. Then, the T cells are genetically modified to become TAA‐specific CAR T cells and administered to the patient by intravenous infusion. The study also assessed the persistence, proliferation and activation of CAR T cells in the peripheral blood of patients.

### Engineered TCR T cell therapy

The most relevant oncoproteins of HR‐HPVs are nuclear and do not show on the surface of the tumour cells. However, they contain epitopes that are presented by MHC molecules and can be recognized by TCRs. Engineered TCR T cells recognizing these epitopes have an advantage over CAR T cells.

T lymphocytes are essential cells of the adoptive immune system to eliminate tumour cells. T cells become activated through interaction between their TCRs and the peptide epitopes loaded on MHC molecules (pMHC). Epitopes from E6 and E7 are processed and presented by MHC class I molecules, and hence can be ideal targets for TCR‐based immunotherapy as they are absent in healthy tissues.^54^

**Figure 2:**
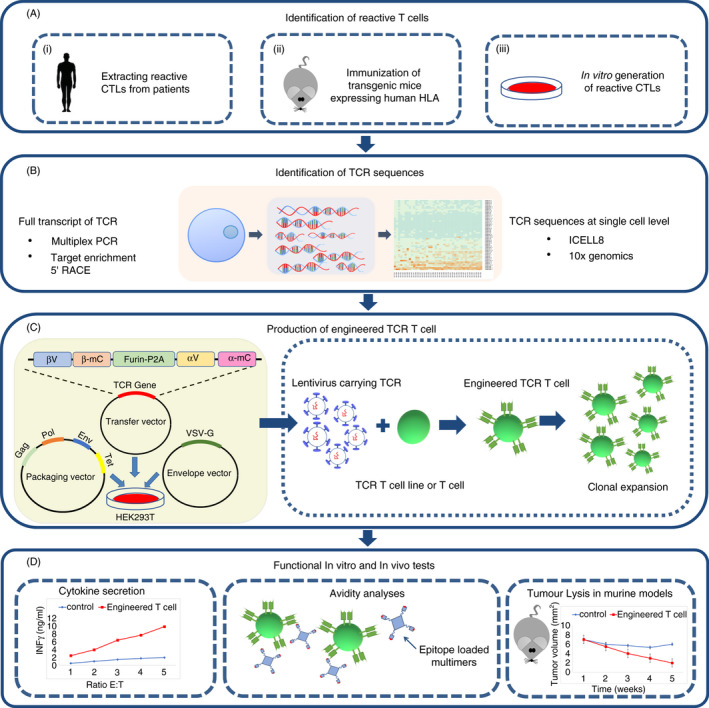
Workflow for the identification of tumour‐specific TCR sequences and for construction of engineered TCR T cells. (A) Reactive CTLs to a specific epitope can be obtained through different strategies: (i) extraction from patients suffering from the tumour under study; (ii) immunization of transgenic mice expressing human HLA (humanized antigen presentation) with a tumour‐specific or tumour‐associated antigen; and (iii) in vitro generation of reactive CTLs by stimulation of PBMCs from healthy donors or patients. (B) The complete sequences of TCRs of reactive CTLs are identified based on entire transcriptome or single‐cell platforms. (C) The candidate TCR genes are engineered to include in tandem: TCR‐β variable (βV)–murine constant region of β chain (β‐mc)–Furin‐P2A–TCR‐α variable (αV)–murine constant region of α chain (α‐mc). Then, naïve T cells or a TCR‐α^−^β^−^ T cell line is transduced with a lentiviral vector expressing the fusion gene to generate engineered TCR T cells that are expanded for further studies. (D) The engineered TCR T cells are then characterized for antigen affinity and avidity and for functionality by in vitro and in vivo assays

Several approaches have been described to identify tumour‐specific T cells: (i) isolation of HPV‐reactive T lymphocytes from patients experiencing tumour regression; (ii) immunization with human oncogenic proteins of transgenic murine models expressing human HLA; and (iii) generation of CTLs by in vitro stimulation of T cells extracted from patients or healthy donors^55^, ^56^, ^57^ (Figure 2A).

After having isolated a population of T cells reactive to the target pMHC, their TCRs are identified by single‐cell sequencing. Several methods, based on cDNA library preparation, provide entire transcripts of the TCR chains: multiplex PCR, target enrichment and 5′ RACE. Furthermore, single‐cell sequencing technologies, such as single‐cell RT‐PCR and paired SEQ, can identify the TCR repertoire at single‐cell level. The ICELL8 single‐cell system of Takara Bio USA, Inc. and 10XGenomics, Inc. also offers platforms for V(D)J analysis at single‐cell level^58^ (Figure 2B). Once HPV‐specific TCRs have been identified and cloned, their TCR‐α and TCR‐β genes are delivered to a TCR‐deficient human T cell line, such as JurkatΔ76, for further characterization. Gene delivery can be achieved by RNA or plasmid DNA transfections, or by using a viral vector.^59^, ^60^, ^61^ Due to its high efficiency and long‐term expression, virus‐mediated delivery is usually preferred, especially using adeno‐associated viruses, gammaretrovirus and lentivirus^62^, ^63^ (Figure 2C). Subsequently, the engineered T cells undergo in vitro and in vivo functionality and avidity tests (Figure 2D).

### Design of lentiviral vectors expressing engineered TCRs

It has been shown that using the murine constant region of TCRs can increase cytokine secretion signals and reduce the chance of mispairing of transgenic and endogenous TCRs.^64^ Moreover, introducing an additional disulphide bond and mutations in the murine constant region to introduce hydrophobic amino acids can enhance structural avidity, that is the binding strength of their TCRs to pMHCs.^65^, ^66^ Sequences encoding the alpha and beta chains are placed sequentially on the same open reading frame and are expressed as a fusion protein. A Furin cleavage site and a self‐cleaving 2A sequence are added between alpha and beta chains. Furin is a member of the proprotein convertase family, which cleaves proteins downstream of basic amino acids such as arginine and lysine.^66^ 2A self‐cleaving peptides (2A peptides) are derived from viruses, and the most prevalent are as follows: P2A, E2A, F2A and T2A. Based on recent studies, P2A, derived from porcine teschovirus 1, is more efficient than others.^67^ For a more effective function, it is recommended that a glycine–serine spacer, such as GSG or SGSG, is included between the Furin cleavage site and the 2A peptides.^68^ A schematic representation of an engineered TCR gene is depicted in Figure 2C.

### Structural avidity characterization of engineered TCR T cells

After having evidence showing that the engineered TCR T cells are able to recognize their target epitope on MHC class I, their structural avidity has to be measured. Nauerth et al^69^ described a method by which the dynamic dissociation (*K*_off_ rate) can be quantitatively measured. This assay is based on reversible Streptamer including an engineered streptavidin, called as Strep‐Tactin^®^, and small peptides with high affinity to Strep‐Tactin (Strep‐tags^®^), which are linked to fluorescently labelled monomers of the epitopes under study.

The binding of engineered TCR T cells to the target cells can be analysed also using the LigandTracer^®^ instrument (Ridgeway Instruments AB). LigandTracer^®^ detects the interaction of labelled molecules with cells in real time. The instrument has an inclined rotating platform on which the dish is placed. On one side of the Petri dish, the target cell is immobilized and pulsed with the desired peptides. The dish is placed on the rotating support, and the background fluorescence is measured. Then, the engineered TCR T cells, labelled with carboxyfluorescein succinimidyl ester, are added to the other side of the Petri dish. The rotation starts, and binding of the T cells to the target cells is measured by subtraction of the fluorescent signal on the side of the target cells from that of the side of the plate without target cells.^70^

Surface plasmon resonance (SPR) is another approach that has been used extensively to analyse TCR‐pMHC interaction.^71^ In this technique, biotinylated pMHC is fixed covalently to a streptavidin‐coupled sensor chip. Biotinylated pMHC loaded with an irrelevant epitope is used as a control. Then, the engineered TCR T cell suspension is injected and flows over the chip. Due to the binding of TCR to pMHC, the intensity of signal results increased.^72^ A more recent system, the Cell Avidity Analyzer z‐Movi™ (Lumicks), uses target cells or ligands immobilized on the surface of a chip through which the T cells are circulated. Then, an acoustic force ramp is applied by which cells are progressively flushed out. Low avidity cells require less force to be separated from their targets. The interaction strength of effector to target cells is measured over time so that each effector cell population is characterized both absolute and relative to other populations by gradually increasing the force.

### Assays for evaluating the function of engineered TCR T cells

The production of cytokines (IFN‐γ, IL‐2 and TNF‐α) upon binding to target cells can be measured as an indication of functional engineered TCR T cells. In these assays, T2 or 293‐A2 cells are pulsed with E7‐derived peptides and co‐cultured with E7‐specific engineered TCR T cells. Detection of these cytokines above background levels (e.g. those obtained with irrelevant peptide) indicates specific activation.^73^, ^74^

The cytolytic activity of T cells has been classically assessed by the chromium (^51^Cr) release assay. However, bioluminescence imaging and real‐time cell assays are non‐invasive and there is no need for prior labelling of the cells. The xCELLigence system is one such assay in which electrical impedance is measured to determine cell viability. The read‐out of such experiments is shown as cell index over time by which the lower the cell index, the higher the lysis rate of target cells.^74^

Measuring transcription factor activity upon TCR‐pMHC engagement can also reveal the activation of T cells. Nuclear factor ‘kappa‐light‐chain‐enhancer’ of activated B cells (NF‐κB), nuclear factor of activated T cells (NFAT) and activator protein 1 (AP‐1) are transcription factors playing important roles in T cell differentiation, proliferation and activation. A triple‐parameter reporter has been derived from Jurkat J76 cells, in which responsive elements for the transcription factors induce the expression of fluorescent proteins.^75^ There are also commercially available T cell activation bioassays for measuring NFAT and the secretion of IL‐2, an indirect sign of NFAT activation, and the read‐out is based on luminescence. Importantly, the engineered TCR T cells should be tested in vivo to confirm their antitumour activity (Figure 2D). Engineered T cells that meet the functional and structural avidity criteria and show efficient tumour eradication in vivo can be then considered as good candidates for clinical trials.

### Clinical trials using engineered TCR T cells targeting HPV+cancers

In an exploratory in vitro study with TILs isolated from tumour fragments of a patient with metastatic HPV16(+) anal cancer, Draper et al. used the TCR engineering approach to retarget T cells against the E6 protein of HPV16.^73^ Using the sequence of the dominant E6‐reactive TCR clonotype, which recognized HLA‐A*02:01/E6_29‐38_ tetramers, genetically engineered T cells were generated from PBMCs of HPV16 (+) cancer patients. Based on this, a phase I/II trial (NCT02280811) was conducted with 12 HPV (+) cancer patients. Two partial responses lasting up to 6 months were reported.

TCRCure Biopharma Ltd introduced another E6‐specific engineered TCR T cell, which is being evaluated in a phase I study with patients with HPV16 (+) cancers (NCT03578406).

Recently, Jin et al.^74^ isolated and sequenced T cell clone from cervix‐infiltrating lymphocytes, which showed high HLA‐A*02:01/E7_11‐19_ tetramer binding and sequenced its TCR. After introducing slight modifications (reversing alpha and beta chain order, adding an extra disulphide bond and several hydrophobic substitutions and cloning into the MSGV1 retrovirus vector), they generated engineered TCR T cells and tested their avidity and efficacy in vitro and in a mouse model. Later on, a phase I/II clinical trial (NCT02858310) was started to determine the safety and efficacy of E7 TCR‐engineered T cells in HPV16‐associated cancers, which is still ongoing. They also evaluate the effect of this E7 TCR T cells for vulvar HSIL in a phase II clinical study (NCT03937791). Furthermore, a recent study has revealed information about the TCR repertoire of cervical cancer patients experiencing tumour remission or failed to clear tumour.^76^ This kind of data can provide a platform for further investigation to find other possible TCR sequences to target HPV‐positive cancers.

## CONCLUSION AND FUTURE PERSPECTIVE

Antibodies against immune checkpoint inhibitors, such as pembrolizumab, have shown antitumour activity in patients with HPV16 (+) PD‐L1‐expressing tumours. However, these inhibitors have to be used in combination with radiotherapy, chemotherapy or therapeutic vaccines. Among therapeutic vaccines targeting HPV16, VGX‐3100 and ADXS11‐001 are in phase III trials, and they could get clinical approval soon. However, most therapeutic vaccines are still in early trial phases. Approaches to combat the immune‐evasive capacity of the tumour microenvironment and improving the trafficking of genetically engineered T cells into tumour tissues are important determinants for successful cell‐based immunotherapies. Due to the complex nature of HPV‐positive carcinoma, combination of different immunotherapeutic approaches will be necessary.

## CONFLICT OF INTEREST

The authors declare that they have no conflict of interests.

## DATA AVAILABILITY STATEMENT

Not applicable.
